# Predictors of the Size of Prosthetic Aortic Valve and In-Hospital Mortality in Aortic Valve Replacement

**DOI:** 10.5539/gjhs.v6n4p177

**Published:** 2014-04-16

**Authors:** Muhammad Shahzeb Khan, Faizan Imran Bawany, Mudassir Iqbal Dar, Muhammad Umer Ahmed, Mehwish Hussain, Mohammad Hussham Arshad, Asadullah Khan

**Affiliations:** 1 Dow Medical College, Dow University of Health Sciences, Karachi, Pakistan; 2 Cardiac Surgery Department, Civil Hospital, Karachi, Pakistan; 3 Ziauddin Medical College, Karachi, Pakistan; 4 Aga Khan University Hospital, Karachi, Pakistan

**Keywords:** aortic valve size, aortic valve replacement, aortic root dimension

## Abstract

**Purpose:**

We hypothesized that gender, age, aortic root dimension, blood group and Left Ventricular End Diastolic and Systolic Diameters may have a significant correlation with the size of mechanical valve used.

**Methods:**

We included 48 patients retrospectively who had been operated at a single tertiary hospital. All patients with aortic stenosis or regurgitation were included in the study. Patients who had undergone previous cardiac surgery or concomitant surgical procedures, such as coronary artery bypass grafting, were excluded from the study.

**Results:**

The median size of the valves used in males (23mm) and females (21mm) were significantly different (P = 0.001). Size of the valve used was significantly associated with Left Ventricular End Systolic Diameter (LVESD) (r = 0.327, P = 0.007) and aortic root dimension (r = 0.526, P < 0.001). Moreover, significantly higher values of LVESD were observed in the expired patients (P = 0.023).

**Conclusion:**

This study shows that aortic root dimension and gender may be important predictors for the size of the prosthetic aortic valve used in aortic valve replacement. Our study also concludes that LVESD has significant relationship with in-hospital mortality. However, more long term clinical trials should be conducted to confirm these relationships.

## 1. Introduction

Aortic valve replacement (AVR) is a procedure that remains the most frequent operation among all the cardiac valve surgeries (Sedrakyan et al., 2004). It is estimated that 13 percent of all cardiac surgeries involve AVR (Cohen, David, Ivanov, Armstrong, & Feindel, 1999). Moreover, around 300,000 valves are implanted globally every year (Pibarot & Dumesnil, 2009). It is approximated that mortality rate of aortic valve disease in 5 years varies from 50 to 80% (Waszyrowski, Kasprzak, Krzeminska-Pakula, Dziatkowiak, & Zaslonka, 1997). The surgery helps to improve the mortality and morbidity significantly in patients with severe aortic valve disease (Kvidal, Bergstrom, Malm, & Stahle, 2000). Hence, AVR remains the treatment of choice in patients having long term aortic valve disease (Libby, Bonow, Mann, & Zipes, 2008).

In our region one of the most common reasons for aortic valve malfunction is Rheumatic Heart Disease (RHD) as a sequel to Rheumatic fever. RHD is a major health challenge particularly in developing countries where it leads to 240,000 deaths annually (Sampaio et al., 2007). About 30-45% of cases of rheumatic fever results in RHD that is characterized by valvular inflammatory infiltrates and Aschoff bodies in myocardium (Sampaio et al., 2007). Recurrent inflammation of valves most commonly results in valvular malfunction.

Patient prosthesis mismatch (PPM) may result in greater pressure gradient across prosthesis and could lead to slow and incomplete reversion of left ventricular hypertrophy (Sim, Orszulak, Schaff, & Shub, 1994) due to aortic stenosis. Outcome of AVR is favorably influenced by valve size and hemodynamic performance (David et al., 1998). Therefore, many of the post operative complications can be avoided by selecting the best possible size and type of the valve. We hypothesized that gender, age, aortic root dimension, blood group, Left Ventricular End Diastolic Diameter (LVEDD) and Left Ventricular End Systolic Diameter (LVESD) may have a significant correlation with the size of mechanical valve used. With this information in mind, cardiac surgeons can easily predict which size of valve to be used in patients having certain demographic and pre-operative variables.

## 2. Materials and Methods

We included 48 patients who had been operated retrospectively at a single tertiary hospital. All patients with aortic valve disease, either stenosis or regurgitation, were included in the study. Patients who had undergone previous cardiac surgery or concomitant surgical procedures, such as coronary artery bypass grafting, were excluded from the study. Left ventricular function and aortic root dimension measurement were evaluated using echocardiography. All other variables used in the data analyses were obtained from the database of the hospital.

Prosthesis selection was done according to the surgeon’s preference. The relationship of prosthesis size to aortic root dimension, gender, blood group, in hospital mortality, LVESD and LVEDD was investigated. This retrospective study was approved by the Institutional Review Board of Dow University of Health Sciences.

Data were entered and analyzed in SPSS version 18.0. Categorical variables were presented in frequencies along with percentages. Continuous variables were expressed as Mean ± standard deviation and Median (Inter-quartile range) for highly skewed variables. Chi-square test was executed for testing association between categorical variables. Normality test for continuous variables was assessed by Shapiro-Wilk’s test and all the variables were found to be skewed. Therefore, Mann Whitney U test and Kruskal Wallis test were performed to compare continuous skewed variables between two and more than two categorical factors respectively. Spearman’s Correlation was computed for finding the association between skewed continuous variables. *P* value less than 0.05 was considered to show significant effect of the variables.

## 3. Results

**Figure 1:**
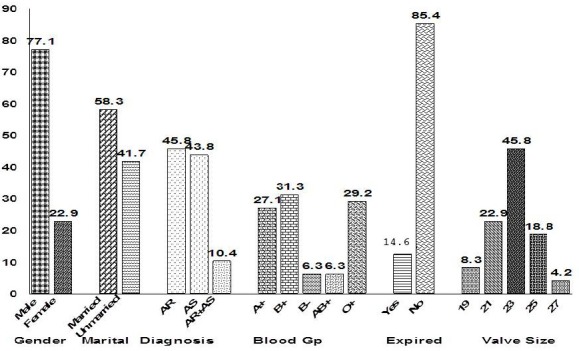
Percentage distribution of demographic and clinical factors

A total of 48 patients were included in the study. Among those, 37 (77.1%) were males and 28 (58.3%) were married. The mean age of the patients was 30.2 ± 14.8 years. Twenty two (45.8%) patients had aortic regurgitation, 21(43.8%) had aortic stenosis and only 5 (10.4%) had both aortic regurgitation and aortic stenosis. The blood group of most of the patients was B+ (n = 15, 31.3%) followed by O+ (n = 14, 29.2%) and A+ (n = 13, 27.1%) (Figure 1).

The mean ejection fraction was 62.0 ± 12.9 %. Most of the patients (n = 22, 45.8%) underwent AVR with a valve size of 23mm. The mean values of LVEDD and LVESD were 56.3 ± 14.7mm and 40.4 ± 10.9mm respectively.

The median size of the valve used in males was 23mm while in females it was 21mm. The size of the valve used in males and females were significantly different (P = 0.001). Most (n = 18, 48.6%) of the valve-used in males were of size 23mm with the smallest and largest one being 21mm (n = 8, 21.6%) and 27mm (n = 2, 5.4%) respectively. The remaining males were implanted with aortic valve of size 25mm (n= 9, 24.3%). On the other hand, females were implanted with only three types of valve sizes; 19mm (n = 4, 36.4%), 21mm (n = 3, 27.3%) and 23mm (n = 4, 36.4%) (Table 1).

**Table 1:** Comparison of valve size with gender

		Male		Female			P Value

		Frequency	Percent	Frequency	Percent		
**Valve Size(mm)**	**19**	0	0.0	4	36.4	
	**21**	8	21.6	3	27.3	
	**23**	18	48.6	4	36.4	0.001
	**25**	9	24.3	0	0.0	
	**27**	2	5.4	0	0.0	

Age (r = 0.224) and LVEDD (r = 0.209) were positively associated with the size of the valve used. Though, these associations were weak and insignificant. However, as the size of the valve used increased, the LVESD (r = 0.327, P = 0.007) and aortic root dimension (r = 0.526, P < 0.001) also increased significantly. A nearby significant positive association was observed between age and aortic root dimension (r = 0.267, P = 0.067). The aortic root dimension was also positively associated with LVESD (r = 0.210, P = 0.152). Ejection fraction had insignificant effect on the size of the valve used. LVEDD and LVESD were directly proportional to each other with strong degree of association (r = 0.807, P < 0.0001). With the increase of LVEDD, the ejection fraction decreased insignificantly (r = -0.270, P = 0.064). However, as the value of LVESD increased, a significant decrement in ejection fraction was observed (r = -0.553, P < 0.0001) (Table 2).

**Table 2:** Correlation of continuous demographic and clinical factors

		Age	Valve Size	LVEDD	LVESD	Ejection Fraction
**Valve Size**	Correlation	0.224	1			
	*P Value*	*0.127*				
**LVEDD**	Correlation	-0.179	0.209	1		
	*P Value*	*0.222*	*0.153*			
**LVESD**	Correlation	-0.149	0.387	0.807	1	
	*P Value*	*0.312*	*0.007*	*<0.0001*		
**Ejection Fraction**	Correlation	0.020	-0.153	-0.270	-0.553	1
	*P Value*	*0.891*	*0.299*	*0.064*	*<0.0001*	
**Aortic Root Dimension**	Correlation	0.267	0.526	-0.013	0.210	-0.101
	*P Value*	*0.067*	*<0.0001*	*0.928*	*0.152*	*0.496*

Out of the 48 patients, seven (14.6%) had in-hospital mortality. The values of LVEDD and ejection fraction were not different in expired and survived patients (P > 0.05). However, significantly higher values of LVESD were observed in the expired patients (P = 0.023). Out of the seven deaths, no death was seen in patients who had both aortic regurgitation and aortic stenosis. Out of 14 patients from O+ blood group, 3 (21.4%) died and out of the 3 patients from AB+ blood group, 2 (66.7%) died. On the other hand, no patients with blood groups B+ and B- had inhospital mortality (Table 3). The odds ratio analysis for patients with blood group B showed that they had 20.7% less chance of mortality as compared to patients with other blood groups (OR = 0.793, 95% CI = 0.659:0.955).

**Table 3:** Comparison of mortality with demographic and operative variables

	Alive		Expired		*P* Value
**Male**	32	86.5%	5	13.5%	0.700
**Female**	9	81.8%	2	18.2%	
**A+**	11	84.6%	2	15.4%	
**B+**	15	100.0%	0	0.0%	0.099*
**B-**	3	100.0%	0	0.0%	
**AB+**	1	33.3%	2	66.7%	
**O+**	11	78.6%	3	21.4%	
**AR**	18	81.8%	4	18.2%	
**AS**	18	85.7%	3	14.3%	0.026*
**AR+AS**	5	100.0%	0	0.0%	
**Age**	29.61 ± 14.20	25 (22)	33.43 ± 18.59	26 (33)	0.598
**LVEDD**	55.90 ± 14.00	55 (20)	58.57 ± 19.47	62 (14)	0.273
**LVESD**	39.10 ± 11.08	38 (14.5)	47.71 ± 6.16	46 (8)	0.023
**Ejection**	62.68 ± 12.98	65 (15.5)	57.71 ± 12.24	58 (13)	0.356

* P Value obtained from Uncertainty Coefficient.

## 4. Discussion

In United States and other western countries, aortic stenosis generally arises as a part of aging process. However, in Pakistan most pathologies of aortic valve arise due to RHD. It is shown by studies that RHD is endemic in Pakistan and is the major cause of premature mortality and disability in the country (Rizvi et al., 2004). Studies have indicated that population of Pakistan belonging to low socio-economic status has a high prevalence of RHD which has not dropped since the last 30 years (Rizvi et al., 2004). Unfortunately, most patients with RHD do not get appropriate medical intervention. Hence, majority of the patients presenting at our cardiac surgery department are already in end stage condition with extremely damaged aortic valves leading to high chances of mortality. Therefore, AVR is an extremely important surgical procedure which can help to improve morbidity and mortality. The success of the surgery largely depends upon durability and size of the prosthetic valve used (Pibarot & Dumesnil, 2009).

However, it is still sometimes difficult for cardiac surgeons to decide the operative procedure and size of prosthetic valve to be used for patients with small aortic root dimension undergoing AVR due to aortic stenosis (Pisano et al., 2012). According to PPM theory of Rahimtoola (1978), using a small prosthetic aortic valve for aortic stenosis causes slow reversal of left ventricular mass, increased long term mortality and increased risk of valve associated complications. PPM is considered positive when the effective orifice diameter is less than 0.85cm^2^/m^2^ (Pibarot & Dumesnil, 2003). In order to prevent PPM, surgeons calculate patient’s body surface area and then determine the valve size required to guarantee an Effective Orifice Area (EOA) of greater than 0.85cm^2^/m^2^. The type and the size of the valve that matches an EOA of greater than 0.85 cm^2^/m^2^ is then chosen for AVR (Pibarot & Dumesnil, 2003). Our study highlights that other variables like LVESD and Aortic Root dimension may also be used to determine the size of valve required for AVR. Our data shows that as the values of LVESD and aortic root dimension increased, the size of the valve required for AVR also increased significantly. Our study also highlighted that greater the age of the patient, greater the aortic root dimension would be. Furthermore, aortic root dimension was also positively associated with LVESD.

In our study age was not found to be a significant predictor of mortality. This is contrary to many studies that have identified age as a significant risk factor for mortality in AVR (Tjang, van Hees, Körfer, Grobbee, & Heijden, 2007). This might be explained by the fact that majority of AVR in our region is due to RHD which normally presents at a much younger age than calcified aortic valve. Our data shows that LVESD can be a very important indicator for in-hospital mortality in aortic valve surgery. This is consistent with a study in which LVESD was independently associated with increased mortality after mitral surgery (Tribouilloy et al., 2009). Other risk factors that have been linked with early mortality in other researches are aortic insufficiency, infective endocarditis, reduced ejection fraction, hypertension, mechanical valve and size of valve used (Tjang et al., 2007). We also recommend that in our region blood group’s impact on mortality should be studied extensively. We found that no death was seen in blood group B patients although most of the patients admitted for surgery were B+. High mortality was observed in AB+ blood group patients.

Interestingly, we also found that gender had a significant impact on the size of prosthetic aortic valve used. However, we feel that our relatively small sample size was a limitation due to which it becomes difficult to generalize all the findings obtained in our research. Additionally our study design was retrospective which has some drawbacks of its own. However, our study has provided a new dimension on which researchers could think and design their studies with larger samples.

This study helps to enlighten the very important matter of choosing a suitable size for the prosthetic aortic valve used. Optimal selection of prosthetic valve in each and every individual patient would lower down the prosthesis related complications drastically. For excellent results, prosthetic valve should mimic the normal valve and should have great implantability which can only be achieved by careful selection of the size of valve used.

## 5. Conclusion

Our study shows that aortic root dimension and LVESD may be important predictors for the size of the prosthetic aortic valve used in AVR. We believe that further researches should be conducted on larger samples to confirm whether an association does exist between aortic root dimension and size of prosthetic aortic valve used. We also conclude that LVESD is a significant predictor for in-hospital mortality of AVR in our setting. However, we recommend that more studies in our region should be conducted to assess more factors associated with mortality. Furthermore, long term clinical trials should also be conducted to determine factors in regard to short, mid and long term mortality.

## References

[ref1] Cohen G, David T. E, Ivanov J, Armstrong S, Feindel C. M (1999). The impact of age, coronary artery disease, and cardiac comorbidity on late survival after bioprosthetic aortic valve replacement. J Thorac Cardiovasc Surg.

[ref2] David T. E, Puschmann R, Ivanov J, Bos J, Armstrong S, Feindel C. M, Scully H. E (1998). Aortic valve replacement with stentless and stented porcine valves: a case-match study. J Thorac Cardiovasc Surg.

[ref3] Kvidal P, Bergstrom R, Malm T, Stahle E (2000). Long-term follow-up of morbidity and mortality after aortic valve replacement with a mechanical valve prosthesis. Eur Heart J.

[ref4] Libby P, Bonow R. O, Mann D. L, Zipes D. P (2008). Braunwald’s Heart Disease: A Textbook of Cardiovascular Medicine.

[ref5] Pibarot P, Dumesnil J (2003). Patient-prosthesis mismatch and the predictive use of indexed effective orifice area: is it relevant?. Cardiac Surg Today.

[ref6] Pibarot P, Dumesnil J. G (2009). Valvular Heart Disease: Changing Concepts in Disease Management. Circulation.

[ref7] Pisano C, D’Amico T, Palmeri C, Franchino R, Fattouch K, Bianco G (2012). Valve prosthesis-patient mismatch: hemodynamic, echocardiographic and clinical consequences. Interact CardioVasc Thorac Surg.

[ref8] Rahimtoola S. H (1978). The problem of valve prosthesis–patient mismatch. Circulation.

[ref9] Rizvi S. F, Khan M. A, Kundi A, Marsh D. R, Samad A, Pasha O (2004). Status of rheumatic heart disease in rural Pakistan. Heart.

[ref10] Sampaio R. O, Fae K. C, Demarchi L. M, Pomerantzeff P. M, Aiello V. D, Spina G. S, Guilherme L (2007). Rheumatic heart disease:15 years of clinical and immunological follow-up. Vasc Health Risk Manag.

[ref11] Sedrakyan A, Hebert P, Vaccarino V, Paltiel A. D, Elefteriades J. A, Mattera J, Krumholz H. M (2004). Quality of life after aortic valve replacement with tissue and mechanical implants. J Thorac Cardiovasc Surg.

[ref12] Sim E. K, Orszulak T. A, Schaff H. V, Shub C (1994). Influence of prosthesis size on change in left ventricular mass following aortic valve replacement. Eur J Cardiothorac Surg.

[ref13] Tjang Y. S, Van Hees Y, Körfer R, Grobbee D. E, van der Heijden G. J (2007). Predictors of mortality after aortic valve replacement. Eur J Cardiothorac Surg.

[ref14] Tribouilloy C, Grigioni F, Avierinos J. F, Barbieri A, Rusinaru D, Szymanski C, Enriquez-Sarano M, MIDA Investigators (2009). Survival implication of left ventricular end-systolic diameter in mitral regurgitation due to flail leaflets a long-term follow-up multicenter study. J Am Coll Cardiol.

[ref15] Waszyrowski T, Kasprzak J.D, Krzeminska-Pakula M, Dziatkowiak A, Zaslonka J (1997). Early and long-term outcome of aortic valve replacement with homograft versus mechanical prosthesis—8-year follow-up study. ClinCardiol.

